# Melatonin Repairs Osteoporotic Bone Defects in Iron-Overloaded Rats through PI3K/AKT/GSK-3*β*/P70S6k Signaling Pathway

**DOI:** 10.1155/2023/7718155

**Published:** 2023-01-17

**Authors:** Maoxian Ren, Hedong Liu, Wenkai Jiang, Zhi Zhou, Xuewei Yao, Zhiyi Liu, Nengfeng Ma, Bing Chen, Min Yang

**Affiliations:** Department of Trauma Orthopedics, The First Affiliated Hospital of Wannan Medical College, Yijishan Hospital, No. 2, Zheshan Xi Road, 241001 Wuhu, Anhui, China

## Abstract

It was found recently that iron overload can cause osteoporosis in rats. Through in vitro and in vivo experimentations, the purpose of the present study was to validate and confirm the inhibitory effects of melatonin on iron death of osteoporosis and its role in bone microstructure improvements. Melatonin (100 mol/L) was administered to MC3T3-E1 cells induced by iron overload *in vitro* for 48 hours. The expression of cleaved caspase-3 and cleaved PARP and the production of ROS (reactive oxygen species) and mitochondrial damage were all exacerbated by iron overload. On the other hand, melatonin restored these impacts in MC3T3-E1 cells produced by iron overload. By evaluating the expression of PI3K/AKT/GSK-3*β*/P70S6k signaling pathway-related proteins (RUNX2, BMP2, ALP, and OCN) using RT-PCR and Western blot, osteogenic-related proteins were identified. Alizarin red S and alkaline phosphatase were utilized to evaluate the osteogenic potential of MC3T3-E1 cells. Melatonin significantly improved the osteogenic ability and phosphorylation rates of PI3K, AKT, GSK-3*β*, and P70S6k in iron overload-induced MC3T3-E1 cells. *In vivo*, melatonin treated iron overload-induced osteoporotic bone defect in rats. Rat skeletal microstructure was observed using micro-CT and bone tissue pathological section staining. ELISA was utilized to identify OCN, PINP, CTX-I, and SI in the serum of rats. We discovered that melatonin increased bone trabecular regeneration and repair in osteoporotic bone defects caused by iron overload. In conclusion, melatonin enhanced the osteogenic ability of iron overload-induced MC3T3-E1 cells by activating the PI3K/AKT/GSK-3*β*/P70S6k signaling pathway and promoting the healing of iron overload-induced osteoporotic bone defects in rats.

## 1. Introduction

The imbalance between the osteogenic and osteoclastic mechanisms leads to the development of osteoporosis. Once bone resorption is higher than bone formation, it reduces bone mineral density and damages the trabecular structure, resulting in a higher risk of bone fracture and brittle bones [1, 2]. People with osteoporosis are more prone to fractures and bone abnormalities [3, 4]. Because of osteoporosis patients' poor bone quality, bone tissue repair is prolonged, causing a more extended recovery period for individuals with bone deformities [5, 6]. Iron content balancing is critical for the efficient functioning of many organ systems [7, 8]. Various disorders, including frequent blood transfusions, hemolysis, osteoporosis, hereditary hemochromatosis, and chronic anemia-*β*-Thalassemia, can cause iron overloads in the body [9]. The homeostasis of iron content and bone tissue are closely related concepts. Through the Fenton reaction, iron excess can generate a lot of ROS that can damage the mitochondria, other organelles, and the DNA and proteins of osteoblasts, alter the dynamic balance of bone tissue, and ultimately induce osteoporosis and other bone abnormalities [10, 11].

Melatonin (MT) is one of the hormones synthesized, stored, and secreted by the pineal gland [12, 13]. Its secretion has a distinct circadian rhythm which is inhibited during the day and promoted at night [14, 15]. Melatonin has been shown in studies to be the most potent endogenous radical scavenger ever discovered [16]. Melatonin's key role is to safeguard cells from oxidative stress damage by acting as an antioxidant [17]. Melatonin can increase the differentiation, mineralization, and proliferation of MC3T3-E1 cells and MSCs (bone marrow mesenchymal stem cells) while reducing their apoptosis [18, 19].

The phosphoinositide 3-kinase/protein kinase B/glycogen synthase kinase 3*β*/p70 ribosomal protein s6 kinase (PI3K/AKT/GSK-3*β*/P70S6k) signaling pathway is an important intracellular signaling pathway that regulates the cell cycle [20, 21]. As a result, it is closely attributed to cell differentiation, mineralization, and proliferation. Melatonin has been found in earlier studies to decrease the oxidative stress caused by arsenic trioxide (As_2_O_3_) via the PI3K/AKT signaling pathway [22]. GSK-3*β* is the downstream substrate and effector of PI3K/AKT [23, 24]. Park et al. reported that high ferric ammonium citrate concentration could inhibit AKT kinase's activity and block AKT/GSK-3*β*/P70S6k signal pathway [25]. However, it remains unclear if melatonin can enhance bone repair of iron overload-induced osteoporotic bone defects and its underlying mechanism in rats.

Therefore, the present research's primary aim was to explore melatonin's impacts on iron overload through *in vivo* and *in vitro* models. Furthermore, this aims to examine how melatonin stimulates the mineralization of iron overload-induced MC3T3-E1 cells via the PI3K/AKT/GSK-3*β*/P70S6k signaling pathway and to investigate if melatonin impacts the repair of osteoporotic bone deformities in iron-overloaded rats.

## 2. Materials and Methods

### 2.1. Grouping Specimen Collection and Processing Methods for *In Vivo* Study

#### 2.1.1. Development of an In Vivo Osteoporosis Model Caused by an Iron Overload

The 8-week-old male Sprague Dawley (SD) rats weighing 210 g (*n* = 70) were obtained for the current study from Nanjing Qinglongshan Animal Breeding Farm, China. Ferric ammonium citrate (FAC, #SLCD3108, SIGMA, Japan) was purchased from Sigma-Aldrich (USA). For a week, the rats were maintained in the animal laboratory with unrestricted access to food and water. The experiment was carried out following the organizational animal care standards. The ethical review committee of Wannan Medical College's First Affiliated Hospital (China) authorized and granted permission for the current project.

After 7 days of environmental adaptation in the animal room, the rats were randomly divided into the CON (intraperitoneal injection: 0 mg/kg/3d) (*n* = 20) and FAC groups (intraperitoneal injection: 100 mg/kg/3d) (*n* = 50) [26]. Rats were injected with FAC every 3 days for 2 months. After 2 months, ten rats were randomly selected from each group to test whether the osteoporosis model was successfully established. Each rat was placed in a metabolic cage for 24 hours before being killed. All soft tissues were extracted from the rat's bilateral femurs, which were subsequently preserved in 10% formalin solution and used for micro-CT scanning and quantification. Afterwards, the bilateral femur of rats was sectioned and analyzed by H&E staining.

#### 2.1.2. Establishment of Osteoporotic Bone Defect Model in Iron-Overloaded Rats

After successfully establishing the osteoporosis model of iron-overloaded rats, the experimental rats underwent bone defect surgery at the lower femur. Specifically, rats requiring bone defect surgery were anaesthetized with pentobarbital sodium (intraperitoneal injection: 40 mg/kg). Then, the lower femur was penetrated from the lateral to the medial condyle with a 1.0 mm diameter Kirschner wire. Penicillin (200000 UI/mL and 1 mL/kg) was intramuscularly injected daily within 3 days after the operation to prevent wound infection. Therefore, it will be used to establish the model of osteoporotic bone defect in iron-overloaded rats.

#### 2.1.3. MT Treatment of Osteoporotic Bone Defect Model in Iron-Overloaded Rats

In the present study, MT was provided by McLean. One week after successfully establishing the osteoporotic bone defect model of iron-overloaded rats, all the animals were divided randomly to one of the following groups: CON group (*n* = 10), FAC group (*n* = 10), FAC+MT group (intraperitoneal injection, 10 mg/kg/d) (*n* = 10), FAC+MT group (intraperitoneal injection, 40 mg/kg/d) (*n* = 10), and FAC+MT group (intraperitoneal injection, 80 mg/kg/d) (*n* = 10) [27]. The FAC+MT group (10 mg/kg/d), FAC+MT group (80 mg/kg/d), and FAC+MT (40 mg/kg/d) group were injected with corresponding doses of MT every day for one month. In order to keep the serum iron content of the rats in the FAC group, FAC+MT (10 mg/kg/d) group, FAC+MT (40 mg/kg/d) group, and FAC+MT (80 mg/kg/d) group unchanged, they will continue to inject FAC intraperitoneally every 3 days for 1 month. The rats in the CON group were given an identical volume of normal saline intraperitoneally.

Each rat was housed in a metabolic cage for 24 hours before being killed, and fasting was required. Anaesthesia was administered with a 40 mg/kg intraperitoneal injection of sodium pentobarbital (CIVI-CHEM, China). A blood collection needle was used to puncture the abdominal aorta to acquire blood samples. The serum was kept at -20°C following a 3000 rpm centrifugation of blood samples for 10 min at 4°C. ELISA (enzyme-linked immunoassay) was then used to detect the bone resorption-related proteins, bone formation-related proteins, and serum iron (SI) in the serum of rats in each group. Bilateral femurs of rats were taken, and all soft tissues were removed, stored in 10% formalin solution, and then used for micro-CT scanning and measurement. After that, the bilateral femurs of rats were sectioned and analyzed by pathological staining.

#### 2.1.4. ELISA Kit Assay

According to the standard operating procedures in the reagent instruction manual, the rat osteocalcin (OCN) ELISA kit (JL21019-48T, JL, Shanghai, China), the rat collagen type I C-terminal peptide (CTX-I), rat procollagen type I N-terminal propeptide (PINP), and rat SI ELISA kits were utilized for each experiment accordingly. We used different kits to detect each group's serum protein and SI of bone defect rats. A microplate reader was then utilized to measure the OD (optical density) at 450 nm. The amounts of OCN, PINP, CTX-I, and SI in the corresponding samples were determined based on the OD value of each sample.

#### 2.1.5. Micro-CT Detection

Following the manufacturer's instructions, *in vitro* micro-CT was utilized to evaluate and categorize the rat's femurs for each group involved in MT treatment of iron-overloaded rat osteoporotic bone defect model trial and osteoporosis model study. Before scanning, the selected scanning area is the lower femur. This region was chosen because it has a well-established bone defect model: the femur was placed into the specimen holder, accommodating up to eight femurs per scan, and the software's operational parameters were selected.

#### 2.1.6. H&E and Masson Staining Kit

In the iron-overloaded osteoporosis model study and the MT treatment of osteoporotic bone defects in iron-overloaded rats, the femurs of rats in each group were submerged in a decalcification solution. The femurs were divided using tissue paraffin sections a month later. The experimental rat femur sections were stained using H&E (hematoxylin and eosin) and a modified Masson three-colour staining kit (#G1346, Solarbio, Beijing, China). An eclipsed Ti-U was used following the staining to observe the bone defect of femur samples in iron-overloaded rats treated with MT and the osteoporosis of femur samples in the iron-overloaded osteoporosis model experimental group.

### 2.2. *In Vitro* Study

#### 2.2.1. Cell Culture

MC3T3-E1 cells (Saiku Biotechnology, Guangzhou, China) were cultured in basic *α*-MEM (minimum essential medium) with 1% penicillin streptomycin solution and 10% FBS (fetal bovine serum) in the current study. At 37°C and 5% CO_2_, the MC3T3-E1 cells were cultured in an FBS medium.

#### 2.2.2. CCK-8 Assay

After 3 days of culture with an *α*-MEM with various concentrations of FAC (0, 100, 200, 400, 800, and 1600 *μ*mol/L), the CCK-8 kit (Cell Counting Kit 8) assay was conducted following the manufacturer's guidelines. The cells were treated for 48 hr with MT (0, 10, 50, 100, 400, and 800 *μ*mol/L). The OD of each well was determined at 450 nm after 1 hour of incubation at 37°C using an enzyme-linked immunosorbent device.

#### 2.2.3. ROS (Reactive Oxygen Species)

ROS detection kit (Beyotime, Beijing, China, #S0033S) was used in this study. To detect ROS in MC3T3-E1 cells, we employed flow cytometry CytoFlex and an inverted fluorescent phase contrast microscope with Ti-U, as specified in the reagent instruction manual, for each group. ImageJ was employed for the quantitative study of ROS.

#### 2.2.4. Mitochondrial Membrane Potential Detection

A mitochondrial membrane potential detection kit (JC-1, #C2006, Beyotime, Beijing, China) was used to measure the level of mitochondrial membrane potential following the manufacturer's instructions. CytoFlex flow cytometry was used for the MC3T3-E1 cells to quantify the mitochondrial membrane potential.

#### 2.2.5. MC3T3-E1 Apoptosis Detection

We used the Annexin V-FITC/PI double staining apoptosis detection kit (BB-4101-50T, BestBio, Shanghai, China) and operated it according to the operating steps of the reagent instruction manual. Flow cytometry FC 500 MPL was used to determine each group's apoptosis rate of MC3T3-E1 cells.

#### 2.2.6. RT-PCR

Following the reagent instruction manual, we used the total RNA extraction kit (#DP424, Tiangen, Beijing, China), super real premix plus (SYBR Green) super real fluorescence quantitative premixed reagent modified version kit, and Fastking cDNA first-strand synthesis kit (DEGenome; KR116-02, Tiangen, Beijing, China). Target mRNA expression in MC3T3-E1 cells was determined using quantitative PCR. The standard 2^*ΔΔ*-CT^ approach was used to calculate the final expression of mRNA. The sequences of all primers utilized for amplification are listed in Table 1.

#### 2.2.7. Western Blot Assay

We extracted total protein samples from the MC3T3-E1 cells cultured in each group using the total protein extraction kit (#P0013B, Beyotime, Shanghai, China). The BCA protein concentration test kit (#P0010S, Beyotime, Shanghai) was utilized to measure the protein concentration. The proteins were separated using 8%, 10%, and 12% sodium sulfate-polyacrylamide gel electrophoresis, depending on their size. Antibodies used in the present study included PI3K (#AF6241, Affinity, USA), p-PI3K (#AF3242, Affinity, USA), AKT (#AF6261, Affinity, USA), p-AKT (Ser473) (#AF0016, Affinity, USA), GSK-3*β* (#AF5016, Affinity, USA), p-GSK-3*β* (Ser9) (#AF2016, Affinity, USA), P70S6k (#AF6226, Affinity, USA), p-P70S6k (Ser371) (#AF3227, Affinity, USA), caspase-3 (#A0214, ABclonal, Wuhan, China), cleaved caspase-3 (#AF7022, Affinity, USA), PARP (#LO7311932, Wanleibio, Shenyang, China), cleaved PARP (#LO7311932, Wanleibio, Shenyang, China), Bax (#AF0120, Affinity, USA), Bcl-2 (#BF9103, Affinity, USA), osteocalcin (#DF12303, Affinity, USA), alkaline phosphatase (ALP, #DF12525, Affinity, USA), BMP2 (#A0231, ABclonal, Wuhan, China), Runx2 (#A2851, ABclonal, Wuhan, China), and *β*-actin (#T0022, Affinity, USA). Using ImageJ software, a quantitative analysis of Western blotting was carried out.

#### 2.2.8. ALP and Alizarin Red S Staining

MC3T3-E1 was divided into CON, FAC, and FAC+MT groups: CON group: after two days, the culture medium was replaced; FAC group: the medium containing FAC (800 *μ*mol/L) was used alternately with the medium without FAC and changed every two days; and FAC+MT group: the medium containing FAC (800 *μ*mol/L) and the medium containing MT (100 *μ*mol/L) were used alternately and changed every two days. On the seventh day, the BCIP/NBT alkaline phosphatase chromogenic kit (#C3206, Beyotime Biotechnology, China) was used for MC3T3-E1 cell staining for each group. Subsequently, ALP expression in MC3T3-E1 cells was determined using an optical camera and eclipsed Ti-U: CON group: obmm osteoblast mineralization medium was changed every two days; FAC group: obmm osteoblast mineralization medium containing FAC (800 *μ*mol/L) was used alternately with obmm osteoblast mineralization medium without FAC and was replaced every two days; and FAC+MT group: obmm osteoblast mineralization medium containing FAC (800 *μ*mol/L) and obmm osteoblast mineralization medium containing MT (100 *μ*mol/L) were used alternately and changed every two days. The MC3T3-E1 cells in each group were stained on the 21st day according to the prescribed protocol using a 0.2% alizarin red S staining solution kit (Beyotime, Shanghai). Afterwards, the optical camera was utilized to observe the osteogenic differentiation of MC3T3-E1 cells in each group and eclipsed Ti-U. Through the ImageJ software, the quantitative analysis of ALP and alizarin red S was carried out.

### 2.3. Statistical Analysis

The statistical analyses were performed using the SPSS 20.0 package. Mean ± SD (standard deviation) was used to represent all quantitative data acquired from at least three biological replicates. Student's *t*-test was used to determine the statistical differences. *P* < 0.05 was used as the criteria for the statistical difference between the groups.

## 3. Results

### 3.1. Iron Overload Causes Osteoporosis in Rats

**Figure 1:**
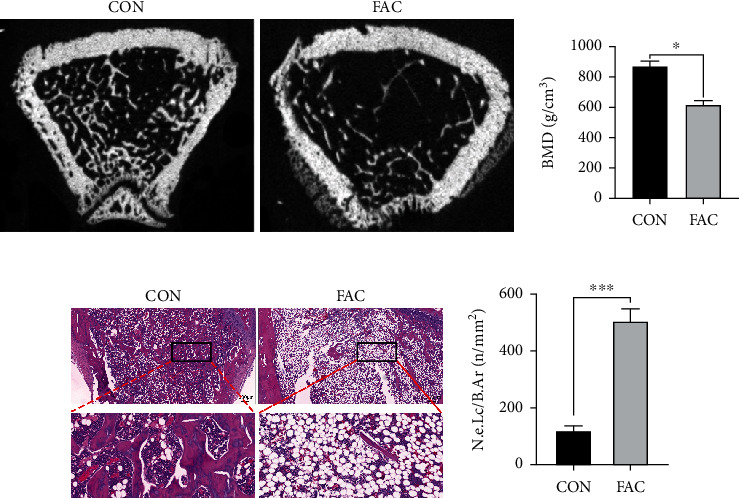
FAC induced osteoporosis in SD rats. (a) Analysis of femoral bone mineral density of SD rats in each group by micro-CT examination. (b) Comparison of BMD of SD rats in each group. (c) Detection of trabecular bone density in SD rats in each group by HE staining. (d) Adipocyte density was compared between the two groups using bone marrow samples from SD rats. N.e.Lc/B.Ar stands for number of empty lacunae per bone area; CON stands for control group; HE stands for hematoxylin-eosin staining; FAC stands for ferric ammonium citrate group; BMD stands for bone density; ^∗^*P* < 0.05 and ^∗∗∗^*P* < 0.001.

We used micro-CT to analyze the femurs of rats in the CON and FAC groups to determine whether iron overload can result in osteoporosis in rats (Figures 1(a) and 1(b)). Rats in the FAC group had lower bone mineral density than those in the CON group. Moreover, we stained the femur of rats in the CON and FAC groups with H&E staining (Figures 1(c) and 1(d)). Rats in the FAC group had lower femoral trabecular bone content and higher bone marrow adipocyte content than the CON group. These findings indicated that iron excess could cause osteoporosis in rats.

### 3.2. MT Reduces the Content of ROS in Iron-Overloaded MC3T3-E1 Cells

**Figure 2:**
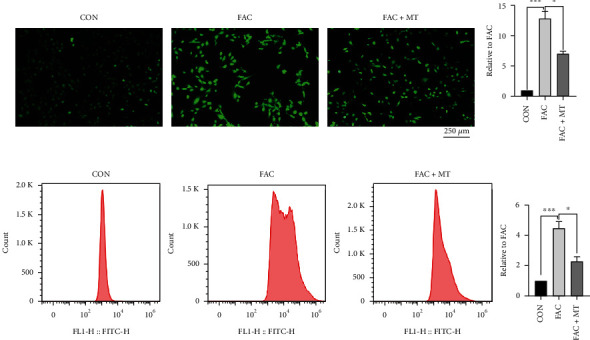
MC3T3-E1 cells that were iron-overloaded. MT decreased ROS levels. (a) The intracellular ROS concentration of MC3T3-E1 cells in each group was discovered using an inverted fluorescent phase contrast microscope (magnification: 100 and scale bar: 250 m). (b) Using flow cytometry to measure the ROS levels in each group's MC3T3-E1 cells. Ferric ammonium citrate plus melatonin is referred to as FAC+MT; ^∗^*P* < 0.05 and ^∗∗∗^*P* < 0.001.

ROS were generated in MC3T3-E1 cells that had been iron-loaded and treated with MT. The ROS level in the MC3T3-E1 cells was lower in the CON group than in the FAC group. ROS levels in MC3T3-E1 cells were lower in the FAC+MT group than in the FAC group (Figures 2(a) and 2(b)). These findings suggested that MT could lower the ROS level in iron-overloaded MC3T3-E1 cells.

### 3.3. MT Promotes Increased Osteogenic Mineralization Capacity and ALP Expression in Iron-Overloaded MC3T3-E1 Cells

**Figure 3:**
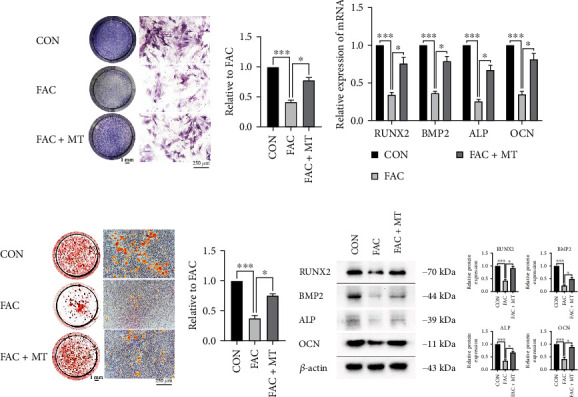
Iron-overloaded MC3T3-E1 cells are more capable of osteogenic mineralization when treated with MT. (a) Alkaline phosphatase expression in MC3T3-E1 cells in each group was determined using an ALP staining. Images obtained from a gross scanner are shown on the left (scale bar: 1 mm), enlarged images are displayed in the middle (magnification: 100 and scale bar: 250 *μ*m), and the quantitative results of the gross scanner images are displayed on the right. (b) ALP, Runx2, BMP2, and OCN mRNA expressions in MC3T3-E1 cells in each group were determined by RT-PCR. (c) Alizarin red S staining was used to identify each group's osteogenic mineralization of MC3T3-E1 cells. Images obtained from a gross scanner are shown on the left (scale bar: 1 mm), magnified images are displayed in the middle (magnification: 100 and scale bar: 250 *μ*m), and a quantitative analysis of the left gross scanning images is displayed on the right. (d) The protein expression of OCN, ALP, Runx2, and BMP2 in MC3T3-E1 cells was determined by using Western blotting. ALP stands for alkaline phosphatase, RUNX2 for runt-related transcription factor 2, OCN for osteocalcin, and BMP2 for bone morphogenetic protein 2; ^∗^*P* < 0.05 and ^∗∗∗^*P* < 0.001.

In order to understand the molecular mechanism of MT-induced osteogenic mineralization, the effect of MT on the osteogenic mineralization of iron-overloaded MC3T3-E1 cells was also studied *in vitro*. The mRNA levels of four osteoblast markers (ALP, Runx2, BMP2, and OCN) were lower in the FAC group than in the CON group in MC3T3-E1 cells. The relative mRNA levels of the four osteoblast markers, OCN, BMP2, Runx2, and ALP, were higher in the FAC+MT group than in the MC3T3-E1 cells of the FAC group (Figure 3(b)). The osteoblast differentiation-related biomarkers, OCN, BMP2, Runx2, and ALP, were less prevalent in MC3T3-E1 cells in the FAC group than in the CON group. ALP, Runx2, BMP2, and OCN expressions in MC3T3-E1 cells from the FAC group were compared to that of the FAC+MT group (Figure 3(d)).

MC3T3-E1 cells were stained with ALP in the CON, FAC, and FAC+MT groups. ALP expression was higher in the FAC+MT group than in the FAC group in MC3T3-E1 cells. ALP expression was lower in the FAC group than in the CON group in MC3T3-E1 cells. Finally, in the CON, FAC, and FAC+MT groups, we stained MC3T3-E1 cells with alizarin red S (Figures 3(a) and 3(c)). For MC3T3-E1 cells, the FAC group demonstrated lower osteogenic mineralization capacity than the CON group. The osteogenic mineralization ability of MC3T3-E1 cells was considerably higher in the FAC+MT group than in the FAC group. The results indicate that MT can promote the restoration of osteogenic mineralization ability in iron-depleted MC3T3-E1 cells by increasing the transcription and expression of osteoblast markers ALP, Runx2, BMP2, and OCN.

### 3.4. MT Decreased the Apoptosis Rate of Iron-Overloaded MC3T3-E1 Cells

**Figure 4:**
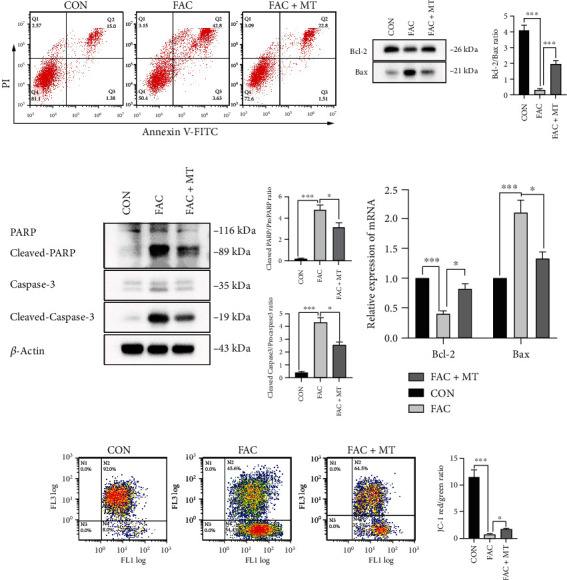
MT decreased the rate of apoptosis in MC3T3-E1 cells that were iron-overloaded. (a) The Annexin V-FITC/PI double labelling technique was used to determine the MC3T3-E1 cells' apoptosis rate in each group. (b) Bax and Bcl2 protein expressions were found using a Western blot analysis. (c) Caspase-3, cleaved PARP, PARP, cleaved caspase-3, and *β*-actin marker expressions were found using a Western blotting in MC3T3-E1 cells between groups. (d) We compared the levels of Bcl-2 and Bax mRNA expressions in MC3T3-E1 cells across treatments using RT-PCR. (e) Each group's MC3T3-E1 cells had their mitochondrial membrane potential measured using flow cytometry. Bcl-2 stands for B-cell lymphoma-2, PARP stands for poly ADP-ribose polymerase, and cleaved PARP stands for cleaved poly ADP-ribose polymerase; ^∗^*P* < 0.05 and ^∗∗∗^*P* < 0.001.

Through the *in vitro* analysis, this study confirmed the MT's antiapoptosis activity in iron-overloaded MC3T3-E1 cells. The early apoptotic ratio of MC3T3-E1 cells was lower in the FAC+MT group than in the FAC group. Compared to the CON group, the fraction of MC3T3-E1 cells that died early increased in the FAC group (Figure 4(a)). The ratio of Bcl-2 to Bax protein expression in MC3T3-E1 cells was lower in the FAC group than in the CON group. In the FAC+MT group, the ratio of Bcl-2 to Bax protein expression in MC3T3-E1 cells was greater than in the FAC group (Figure 4(b)). The ratio of Bcl-2 to Bax mRNA expression in MC3T3-E1 cells was higher in the FAC+MT group than in the FAC group. The ratio of Bcl-2 to Bax mRNA expression in MC3T3-E1 cells was higher in the CON group than in the FAC group (Figure 4(d)).

In the CON group, the ratios of cleaved caspase-3/caspase-3 and cleaved PARP/PARP proteins in MC3T3-E1 cells were lowered compared to the FAC group. In MC3T3-E1 cells, FAC+MT reduced the ratio of cleaved caspase-3/caspase-3 and cleaved PARP/PARP proteins compared to the FAC group (Figure 4(c)). We utilized a mitochondrial membrane potential detection kit to identify and assess the difference in mitochondrial membrane potential between each group of MC3T3-E1 cells (Figure 4(e)). Compared to the CON group, MC3T3-E1 cells in the FAC group had reduced potential for the mitochondrial membrane. The mitochondrial membrane potential of MC3T3-E1 cells in the FAC+MT group was greater than that of the FAC group. These findings suggest that MT regulates iron excess MC3T3-E1 cell apoptosis via Bcl-2 and caspase-3 transcription and expression.

### 3.5. Through the PI3K/AKT/GSK-3*β*/P70S6k Signaling Pathway, MT Regulates the Iron Overload in MC3T3-E1 Cells

**Figure 5:**
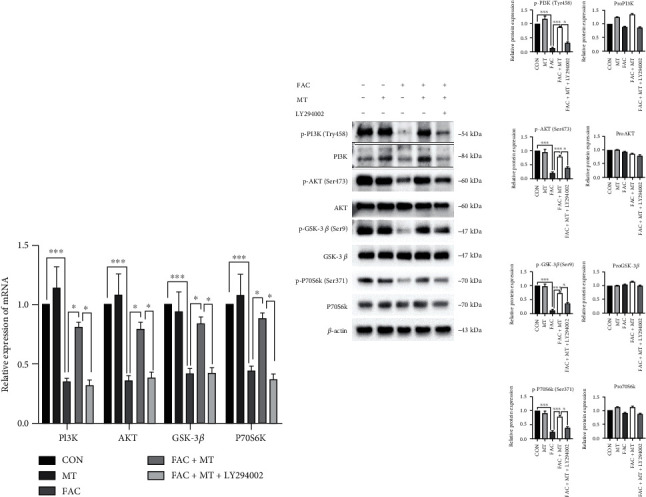
Showing how MT uses the PI3K/AKT/GSK-3*β*/P70S6k signaling pathway to treat iron-overloaded MC3T3-E1 cells. (a) All mRNA expression levels in MC3T3-E1 cells were determined by using RT-PCR for p-PI3K, p-GSK-3*β*, p-AKT, and p-P70S6k. (b) Western blotting was used to analyze the expression of PI3K, AKT, GSK-3*β*, P70S6k, p-PI3K, p-AKT, p-GSK-3*β*, p-P70S6k, and *β*-actin proteins in MC3T3-E1 cells across treatment groups. MT stands for melatonin group; FAC+MT+LY294002 stands for the ferric ammonium citrate+melatonin+LY294002 group; PI3K stands for phosphatidylinositol kinase; p-PI3K stands for phosphorylated phosphatidylinositol kinase; AKT stands for protein kinase B; p-AKT stands for phosphorylated protein kinase B; p-GSK-3*β* stands for phosphorylated glycogen synthase kinase-3*β*; GSK-3*β* stands for glycogen synthase kinase-3*β*; P70S6 stands for ribosomal S6 protein kinase; p-P70S6k stands for phosphorylated ribosomal S6 protein kinase; ^∗^*P* < 0.05 and ^∗∗∗^*P* < 0.001.

We identified and examined the transcription and expression of PI3K/AKT/GSK-3/P70S6k signaling pathway-related genes in each set of MC3T3-E1 cells to determine how MT suppresses apoptosis in iron-overloaded MC3T3-E1 cells. The transcription of the PI3K, AKT, GSK-3*β*, and P70S6k genes in MC3T3-E1 cells was reduced in the FAC and FAC+MT+LY294002 groups compared to the FAC+MT group (Figure 5(a)). In MC3T3-E1 cells, the FAC group and FAC+MT+LY294002 group had lower levels of PI3K (Try458), P70S6k (Ser371), AKT (Ser473), and GSK-3*β* (Ser9) protein phosphorylation than the FAC+MT group (Figure 5(b)). The data suggest that MT modulates the proliferation of iron-overloaded MC3T3-E1 cells via the PI3K/AKT/GSK-3*β*/P70S6k signaling pathway.

### 3.6. MT Can Promote Trabecular Bone Regeneration in Iron-Overloaded Rats

**Figure 6:**
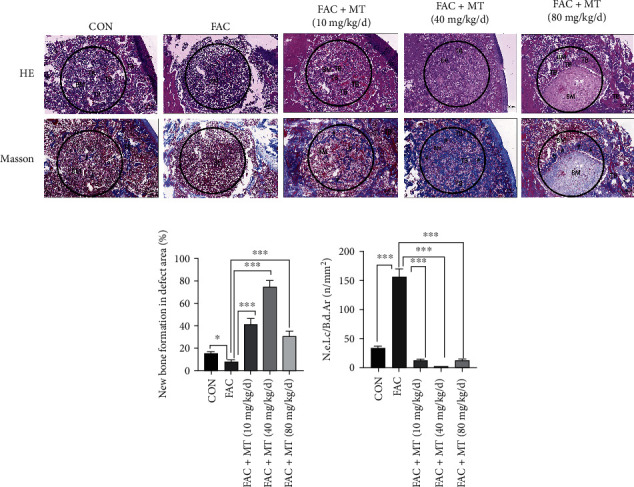
In iron-overloaded SD mice with osteoporotic bone abnormalities, melatonin stimulates trabecular bone repair while decreasing bone marrow adipocyte density. (a) Chemical labeling of nascent bone trabeculae and adipocytes in the bone marrow of SD rats. (b) Rats were divided into two groups, and the pace at which new bone formed in the defect location was compared using the standard deviation. (c) Dissecting the differences between normal and SD rats' bone marrow adipocyte densities in relation to the abnormality's location. Masson staining; N.e.Lc/B.d.Ar, the ratio of the number of empty lacunae to the total bone defect area; significance level for the amount of new bone trabeculae (^∗^*P* < 0.05 and ^∗∗∗^*P* < 0.001). TB = trabecular bone; BM = bone marrow cavity.

We used H&E and Masson staining for the femoral tissues of the rats in each group to understand better the impact of MT on the quantity of trabecular bone and bone marrow adipocytes in the defect area of iron-overloaded animals (Figure 6(a)). Rats in the FAC+MT (40 mg/kg/d) group had a faster rate of new bone formation in the area of the femoral defect than rats in the FAC group (Figure 6(b)). The FAC+MT (40 mg/kg/d) group had less bone marrow adipocytes in the femoral defect area of the rats than the FAC group (Figure 6(c)). In iron-overloaded rats, the present findings revealed that MT could accelerate trabecular bone development while inhibiting the differentiation of bone marrow mesenchymal stem cells into bone marrow adipocytes.

### 3.7. MT Promotes Bone Repair in the Area of Osteoporotic Bone Defect in Iron-Overloaded Rats

**Figure 7:**
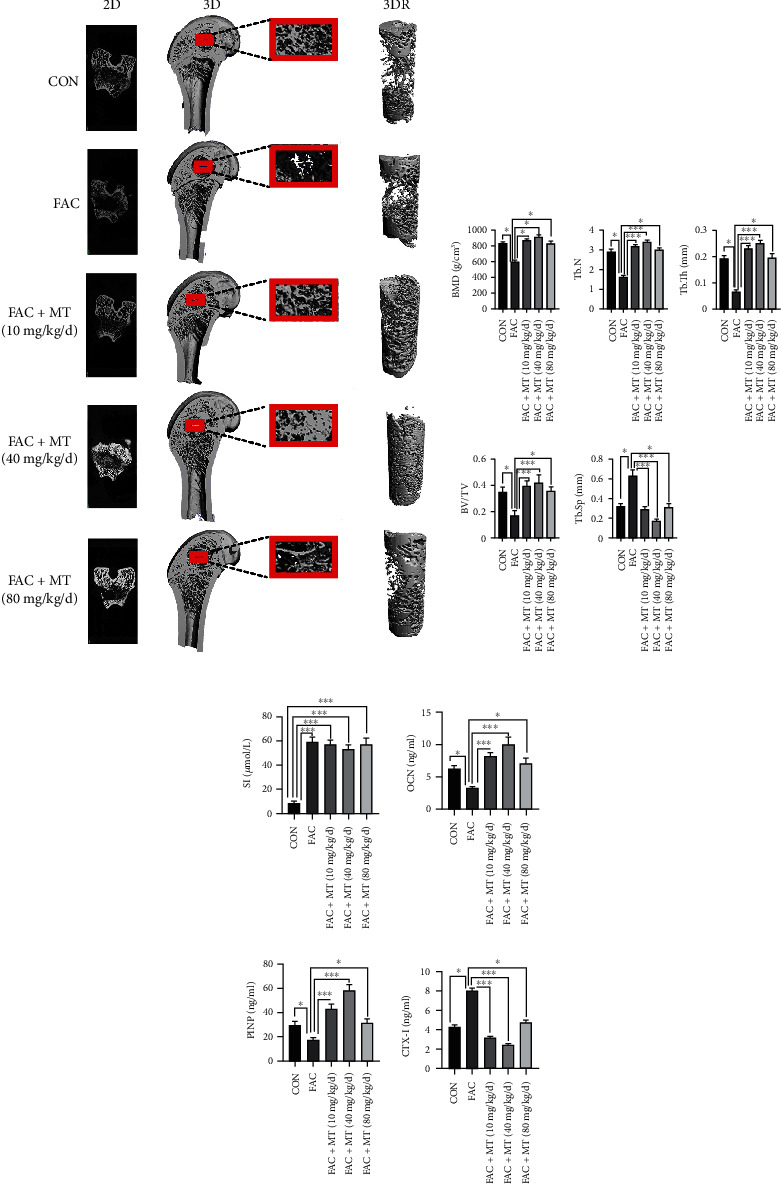
In rats with iron overload and osteoporotic bone abnormalities, melatonin promotes bone healing. (a) By using micro-CT imaging, the bone defect area of SD rats in each group was examined. (b) Comparison of BV/TV, BMD, Tb.Th, Tb.N, and Tb.Sp in each group in the location of the femoral defect in SD rats. (c) Comparison of each group's SD rat's serum levels of SI, PINP, OCN, and CTX-I. 3DR stands for three-dimensional reconstruction; 2D stands for two dimensions; BV/TV stands for bone volume/total mass; Tb.Th stands for trabecular thickness, Tb.N for trabeculae number, and Tb.Sp for trabecular space. SI stands for serum iron; PINP stands for type I procollagen N-terminal propeptide; CTX-I stands for type I collagen C-terminal peptide and OCN for osteocalcin; ^∗^*P* < 0.05 and ^∗∗∗^*P* < 0.001.

In each group of rats for the identification of femurs, the micro-CT was utilized to confirm the role and mechanism of MT in the treatment of osteoporotic bone defects in iron-overloaded rats. In addition, a three-dimensional reconstruction of the defect area at the lower end of the femur was conducted (Figures 7(a) and 7(b)). Rats in the FAC+MT (40 mg/kg/d) group showed higher BV/TV, Tb.Th, Tb.N, and BMD of the femoral defect area and lower Tb.Sp than rats in the FAC group.

Moreover, we detected the differences in SI, OCN, PINP, and CTX-I contents in the serum of rats in each group by ELISA (Figure 7(c)). The serum OCN and PINP levels were more significant in the FAC+MT (40 mg/kg/d) group than in the FAC group, although the CTX-I levels were lower in the FAC group. The SI content of rats in the FAC group, FAC+MT (10 mg/kg/d) group, FAC+MT (40 mg/kg/d) group, and FAC+MT (80 mg/kg/d) group was elevated compared to the CON group. These findings suggested that MT can repair osteoporotic bone defect regions in iron-overloaded rats via modulating serum bone resorption and bone morphogenetic protein levels.

## 4. Discussion

In recent years, due to hemolysis, anemia, and the use of iron supplements, the iron content in the patient's body is getting higher and higher [28, 29]. In the biological organic body, iron is a crucial trace element that can contribute to the formation of haemoglobin [30, 31]. Moreover, people with osteoporosis in clinics have an iron imbalance associated with poor bone health [32]. Previous studies have shown iron overload to block the PI3K/AKT signaling pathway, decrease the AKT kinase activity in MC3T3-E1 cells (osteoblast precursor cells), and ultimately result in MC3T3-E1 cell death [33, 34]. The liver is the hub of metabolic processes and plays a vital role in detoxifying waste metabolites [35]. Ghasemi et al. discovered that iron overload decreases the activity of antioxidant enzymes in the liver, causing the liver to create a significant concentration of ROS and impairing liver function [36]. Vetuschi et al. showed that iron overload could antagonize ferroptosis through negative feedback regulation of hepatic release of glutathione peroxidase 4 [37]. Through its potential to eliminate free radicals, melatonin exhibits its antioxidant actions and protects the liver from oxidative stress [38].

Osteoporosis is a widespread bone disease that develops when osteoblast activity decreases and osteoclast activity increases. This further increases bone fragility, making bones more likely to fracture, destroying bone microstructure, and reducing bone mineral density and quality [39, 40]. While there are two types of osteoporosis, primary and secondary [41], bone marrow adipocytes and osteoblasts also develop from MSCs [42]. The quantity of MSCs that have differentiated into osteoblasts would decrease due to the aberrant rise in adipocytes in the bone marrow [43]. There are also reports that iron overload can cause osteoporosis in rats [26]. However, how to treat osteoporotic bone defects caused by iron overload is not clear. A recent study reported that iron overload causes rats to lose more trabecular bone and have more adipocytes in their bone marrow. For the first time, we discovered that MT prevented MSCs from differentiating into bone marrow adipocytes and promoted the formation of trabecular bone in the bone defect area of iron-overloaded rats. Iron excess induces osteoporosis in rats by promoting the transformation of MSCs into adipocytes. In iron-overloaded rats, MT promotes the growth of trabecular bone in the bone defect area by inhibiting the differentiation of MSCs into bone marrow adipocytes and the serum bone resorption protein and increasing the content of serum bone morphogenetic protein.

Apoptosis is the autonomous and orderly death of cells controlled by genes, which is the crucial mechanism for regulating the stability of an intracellular environment [44]. Studies have shown that iron overload reduces the biological activity of osteoblasts in a concentration-dependent way by increasing apoptosis and reducing proliferation in HFOB1.19 cells due to oxidative stress [45, 46]. Caspase-3 activation is thought to be indicated by cleaved PARP [47]. In this work, the MC3T3-E1 cell's apoptotic rate induced by iron overload was dramatically enhanced, as was the expression of cleaved caspase-3 and cleaved PARP. In MC3T3-E1 cells with an excess of iron, MT repaired these impacts.

Moreover, ROS causes apoptosis by causing organelle damage [48]. Recently, elevated levels of FAC-inducing MC3T3-E1 cell apoptosis through the ROS pathway have been reported [49, 50]. The current investigation found an apparent reduction of ROS levels in MC3T3-E1 cells with iron overload after treatment with MT. These results indicated that MT suppresses apoptosis in iron-overloaded MC3T3-E1 cells via the caspase-3 pathway.

The mitochondrion is the central place for cells to carry out aerobic respiration [51, 52]. According to studies, the apoptotic process causes the mitochondrial outer membrane to become more permeable to a range of proteins present in the mitochondrial membrane gap, causing lethal proteins to enter the cytoplasmic matrix and encouraging apoptosis [53, 54]. According to previous studies, the mitochondrial apoptosis signaling pathway is regulated by Bax and Bcl-2 [55, 56]. Reduced Bcl-2 expression increases protein permeability in the mitochondrial membrane gap, allowing cytochrome C to enter the mitochondrial matrix through the mitochondrial membrane gap and cause it to be released [57, 58].

Cytochrome C can activate the caspase family of apoptotic proteases, leading to MC3T3-E1 cell death. Iron-overloaded MC3T3-E1 cells had a reduced mitochondrial membrane potential, lower levels of the antiapoptotic protein Bcl-2, and higher levels of the proapoptotic protein Bax, according to the current observations. In the iron-overloaded MC3T3-E1 cells, MT administration enhanced the level of mitochondrial membrane potential, increased the expression of the antiapoptotic protein Bcl-2, and lowered the proapoptotic protein expression Bax. These findings suggest that MT controls the amounts of Bcl-2 family proteins to restore the mitochondrial activity of MC3T3-E1 cells in iron-overloaded.

According to previous research, activating the PI3K/AKT signaling pathway regulates osteoblast proliferation and differentiation [59, 60]. In iron-overloaded MC3T3-E1 cells following MT treatment, we discovered that the expression level of phosphorylated PI3K, AKT, GSK-3*β*, and P70S6k protein kinases was much higher, indicating that MT activated the PI3K/AKT/GSK-3*β*/P70S6k signaling pathway in MC3T3-E1 cells. Several earlier reports indicated that LY294002 is an inhibitor of the PI3K/AKT signaling pathway [61]. According to previous studies, PI3K/AKT kinase phosphorylation can inactivate Bax and depolymerize Bax with Bcl-2 [62, 63]. Additionally, MT inhibited the release of cytochrome C from mitochondria, stopped the caspase family of apoptotic proteases from activating, and prevented MC3T3-E1 cells from undergoing apoptosis [64]. According to our findings, the expression of osteogenic genes rose dramatically in iron-overloaded MC3T3-E1 cells treated with MT. MT, an osteogenic agent, has previously been demonstrated to increase the proliferation, mineralization, and differentiation of MC3T3-E1 cells and bone marrow stromal cells (BMSCs) [65]. The osteogenic mineralization ability of iron-overloaded MC3T3-E1 cells was increased after treatment with MT, showing that MT promoted mineralization of iron-overloaded MC3T3-E1 cells via the PI3K/AKT/GSK-3*β*/P70S6k signaling pathway.

There are certain limitations to the current study. The complicated interactions between bone resorption by osteoclasts and bone formation by osteoblasts and the capacity of BMSCs to mineralize bone are the causes of osteoporosis. Nevertheless, we only looked at melatonin's impact on osteoblasts with excessive iron. We will concentrate on this issue in our future research.

## 5. Conclusion

In conclusion, our study demonstrated that MT promotes osteoporotic bone defect healing in iron-overloaded mice and the mineralization, differentiation, and proliferation of iron-overloaded MC3T3 cells via the ROS-PI3K/AKT/GSK-3*β*/P70S6k pathway. Considering our data, we believe that it is necessary to make a human intervention and exogenous supplements of MT content in patients with osteoporosis to achieve the purpose of better antiosteoporosis.

## Figures and Tables

**Table 1:** Sequences of primers used for quantitative PCR.

Primer name	Primer sequences (5′–3′)
PI3K F	TGGGACCTTTTTGGTACGAGA
PI3K R	AGCTAAAGACTCATTCCGGTAGT
AKT F	CCTTTATTGGCTACAAGGAACGG
AKT R	GAAGGTGCGCTCAATGACTG
GSK-3*β* F	ATGGCAGCAAGGTAACCACAG
GSK-3*β* R	TCTCGGTTCTTAAATCGCTTGTC
P70S6k F	CATCGGCACCACTTCCAATA
P70S6k R	TTCATACGCAGGTGCTCTGG
RUNX2 F	TTCAACGATCTGAGATTTGTGGG
RUNX2 R	GGATGAGGAATGCGCCCTA
BMP2 F	TTGGACACCAGGTTAGTGAATCA
BMP2 R	TCTCCTCTAAATGGGCCACTT
ALP F	GAGTCGGACGTGTACCGGA
ALP R	TGCCACTCCCACATTTGTCAC
OCN F	CTCTCTGACCTCACAGATGCCAAG
OCN R	GGACTGAGGCTCCAAGGTAGC
Bax F	AGACAGGGGCCTTTTTGCTAC
Bax R	AATTCGCCGGAGACACTCG
Bcl-2 F	GTCGCTACCGTCGTGACTTC
Bcl-2 R	CAGACATGCACCTCCCCAGC
GAPDH F	TGACCTCAACTACATGGTCTACA
GAPDH R	CTTCCCATTCTCGGCCTTG

## Data Availability

On request, the data described in this article can be acquired from the corresponding author.
